# The epidemic of human papillomavirus virus-related oropharyngeal cancer: current controversies and future questions

**DOI:** 10.1186/s13027-024-00616-0

**Published:** 2024-11-28

**Authors:** Allen M. Chen

**Affiliations:** 1 Department of Radiation Oncology, Chao Family Comprehensive Cancer Center, University of California-Irvine, School of Medicine, Irvine, CA 92617 USA; 2 Department of Radiation Oncology, University of California, Irvine, School of Medicine, Orange, CA 92868 USA

**Keywords:** HPV, Oropharynx, Cancer, Radiation, De-escalation

## Abstract

The incidence of human papillomavirus (HPV) associated oropharyngeal cancer has increased to epidemic-like proportions in the United States and other industrialized nations. While significant progress has been made in the understanding of this disease with respect to its underlying biology and clinical behavior, numerous questions persist regarding treatment. It is now firmly established that patients with HPV-positive oropharyngeal cancer have a significantly improved prognosis as a result of their exquisite radiosensitivity compared to their HPV-negative counterparts and thus can be targeted with de-escalated approaches using reduced doses of radiation and/or chemotherapy. The fundamental goal of de-escalation is to maintain the high cure and survival rates associated with traditional approaches while reducing the incidence of both short- and long-term toxicity. Although the exact reason for the improved radiosensitivity of HPV-positive oropharyngeal carcinoma is unclear, prospective studies have now been published demonstrating that de-escalated radiation can successfully maintain the high rates of cure and preserve quality of life for appropriately selected patients with this disease. However, the selection criteria and specific means for de-escalation remain uncertain, and paradigms continue to evolve. Given that HPV-positive oropharyngeal cancer is increasingly recognized as a public health problem, the search for answers to many of these provocative questions has important societal implications and is the subject of this review.

## Introduction

The worldwide incidence of human papillomavirus (HPV)-associated oropharyngeal squamous cell carcinoma has risen dramatically in recent years reaching epidemic-like proportions in developed countries. With the increased recognition of the role that HPV infection plays in the oncogenesis of oropharyngeal cancer, significant advances have been made over the last several decades in the understanding of this disease. Most strikingly, it is now firmly established that HPV-positive oropharyngeal cancer represents a clinically and biologically distinct entity from HPV-negative oropharyngeal cancer. From a practical standpoint, the background from which HPV-positive oropharyngeal cancer emerges, as well as the risk factors for its development is uniquely different and leads to new questions regarding prevention and treatment. With respect to the latter, radiation therapy, either delivered as primary treatment or postoperatively, is recommended. Historically, this regimen has consisted of 6 to 7 weeks of daily radiation to relatively high doses, often combined with cisplatin chemotherapy. However, this treatment is notoriously difficult to tolerate, and a significant proportion of patients experience acute side effects, most commonly related to irritation and/or ulceration of the mouth and throat. Furthermore, high-dose radiation has been associated with such post-treatment sequelae as dysphagia, xerostomia, neuropathy, and/or neck fibrosis, among others. Unfortunately, the effect of these side effects on quality of life can be severe, life-altering, and permanent in many survivors. For these reasons, considerable research has been focused on identifying customized approaches to treatment based on the presence of the HPV biomarker. While progress has been achieved, numerous questions remain unanswered, and the need to refine these processes define the goals of future research.

## How common is human papillomavirus (HPV)-positive oropharyngeal cancer?

The incidence of HPV-positive oropharyngeal (throat) cancer has been shown to vary across the world, where it is more common in developed, industrialized countries and in Western societies. These discrepancies may be due to differing social norms, particularly with regard to sexual activity. In the United States, approximately 20,000 new cases are diagnosed annually, and current projections estimate a steep upward trend for the next decade with greater than 30,000 annual cases by year 2029 [1]. The overall annual incidence rate is approximately 5 cases per 100,000 in the United States with most new cases arising in white males aged 65 and younger, where it represents the sixth most common non-cutaneous solid cancer. Notably, the incidence of HPV-positive oropharyngeal caner has increased dramatically over the past two decades- with some studies demonstrating a three-fold increase between 2000 and 2017 [2]. In fact, in the United States, oropharyngeal cancer has now eclipsed cervical cancer as the most neoplasm associated with HPV infection.

## Who gets HPV-positive oropharyngeal cancer?

In the United States, the incidence of HPV-positive oropharyngeal cancer differs not only by sex but also by age and race. For instance, a higher prevalence of this disease has consistently been observed in white men than in any other subgroup. Furthermore, patients with HPV-positive oropharyngeal cancer have been shown to be relatively younger (of middle-age) and healthier compared to HPV-negative patients [3]. While smokers can definitely get HPV-positive oropharyngeal cancer, many patients with this disease have never smoked. In parallel with the increased incidence in white men in the USA, higher socioeconomic status is also associated with an increased incidence [4]. In short, an epidemic appears to have developed affecting younger middle-aged men of professional classes.

## What are the risk factors for HPV infection?

HPV is a sexually transmitted disease. Oral HPV is transmitted to the mouth by oral sex, or possibly in other ways. While many people are exposed to oral HPV in their life, only a small minority of these will develop cancer. Population-based data has shown that small proportion of the public harbor the virus at any given time orally and are asymptomatic and/or unaware of this infection. Prospective data from the National Health and Nutrition Examination Survey in which asymptomatic subjects between the ages of 14 and 69 years were screened at mobile examination centers using polymerase chain reaction and in situ hybridization following a 30-second oral rinse and gargle with mouthwash is particularly instructive [5]. In this cross-sectional study, oral HPV infection occurred in a bimodal pattern with respect to age, with peak prevalence at ages 30 to 34 years and 60 to 64 years. The prevalence of any oral HPV infection among men and women were 10% and 4%, respectively. Notably, oral HPV infection was exceedingly rare among those without any type of sexual contract (1%). A strong correlation was identified on multivariate analysis between oral HPV infection and the number of sexual partners. Further, the relationship between oral HPV infection with lifetime number of any sex partners appeared stronger for high-risk subtypes typically associated with oncogenesis including HPV-16 and HPV-18 than for low-risk HPV infections. Although nearly all people clear the virus within 1 to 2 years, HPV infection can persist in some placing them at higher risk for cancer [6].

## How does HPV cause cancer?

HPV is estimated to cause 70% of oropharyngeal cancers in the United States [7]. In some patients with persistent and longstanding infection, HPV can trigger an activation of molecular pathways that lead to cancer. This generally occurs many years after initial exposure, and it is poorly understood why some patients develop cancer and others do not. The exact mechanism of HPV-related oncogenesis is still unclear but is thought to be related to the virus’s ability to infect and persist in epithelial cells by altering their regulation.

HPV is a small DNA virus with a diameter of approximately 55 nm and harboring nearly 8,000 base pairs. The viral genetic material is partitioned into distinct segments: an early (E) section, a late (L) segment and a lengthy control region (LCR). The E region consists of eight genes, and the L region contains two genes. These genes are responsible for the production of viral proteins, whereas the LCR functions as an upstream non-coding regulatory area, accommodating the site for the initiation of viral DNA replication and transcriptional regulatory elements. The virus is well known to infect various epithelial tissues including the epidermis (cutaneous types) and the epithelial linings of the upper respiratory system and anogenital tract (mucosotropic types). The difference in their ability to promote malignant transformation is the basis for the classification of HPV into low- and high-risk subtypes.

Most studies suggest that HPV exerts its oncogenic potential through the expression of the E6 and E7 genes, which generate the oncoproteins E6 and E7 [8]. These oncoproteins inhibit the function of the p53 and Rb proteins, respectively, thereby conferring oncogenic properties to the virus. E6 also interacts with various PDZ proteins, contributing to the disruption of cellular adhesion and polarity. Additionally, E7 interacts with a range of other cellular proteins, including cyclins, cyclin-dependent kinases, and histone deacetylases, facilitating the viral replication and cellular transformation necessary for HPV-associated tumorigenesis. While other viral proteins and pathways have also been shown to contribute to genomic instability and oncogenesis, most studies have focused on the central role of E6 and E7 given their ability to disrupt known tumor suppressor pathways [9]. Notably, oropharyngeal and cervical cancer are not the only sites where HPV has been shown to play a role in causing cancer. For instance, HPV has been implicated as a causative agent for cancers arising from the anus, vagina, and vulva.

## Can HPV vaccination prevent and/or treat oropharyngeal cancer?

The Centers for Disease Control and Prevention currently recommends routine vaccination against HPV of boys and girls at the age of 11–12 years [10]. For children who receive their first dose before the age of 15, only two doses are necessary. Teens and young adults who start the series later, at ages 15 through 26 years, need three doses of HPV vaccine. Notably, HPV vaccination is not recommended for anybody older than age 26 years because the likelihood of being exposed to HPV is extremely high. Vaccination is generally protective against all HPV-associated cancers including those of the oropharynx. Notably, vaccination can prevent cancer, but it cannot treat. In other words, it does not work against established HPV infections or against cells that have been transformed by HPV and are on their way to forming tumors. However, novel, therapeutic vaccinations directed against HPV oncoproteins are under development; the results from early-stage clinical trials are eagerly anticipated and could have clinical implications in the future.

## What are the symptoms of HPV-positive oropharyngeal cancer?

Since the oropharynx is the middle part of the pharynx (throat and back of tongue) behind the mouth, common symptoms of cancer arising from the region include sore throat, pain or difficulty with swallowing, hoarseness, trouble opening the mouth or speaking, ear pain, unexplained weight loss, and coughing up of blood. In some patients, the finding of a new lump in the neck is the first sign of cancer.

## What is the prognosis for this disease?

Evidence has now firmly established that HPV-positive oropharyngeal cancer is a unique entity with distinct clinical and molecular characteristics compared to HPV-negative oropharyngeal cancer. Data has accumulated demonstrating that patients with HPV-positive oropharyngeal cancer have a significantly improved prognosis as a result of their exquisite sensitivity to radiation compared to their HPV-negative counterparts [11–14]. In other words, these tumors have been shown to shrink briskly and robustly in both the laboratory and in actual patients.

The evidence suggesting that HPV imparts radiosensitivity to oropharyngeal tumors is compelling. In vitro data analyzing survival fraction after a single exposure of 10 Gy has shown significantly lower rates for HPV-positive cell lines than for HPV-negative cell lines [12]. Additionally, the levels of γ-H2AX foci formation, a measure of radiation-induced double-strand DNA breaks, and retention were time and cell line-dependent. For the HPV negative cell lines, the γ-H2AX levels started to increase at 1 h and peaked at 4 h after radiation before reducing to negligible levels. In contrast, the HPV-positive cell lines displayed persistent γ-H2AX activity with the expression of γ-H2AX remaining high even at 48 h post radiation. Mouse models have also been developed demonstrating the ability of HPV to confer radiosensitivity in vivo [13, 14].

Moreover, given the strong link between HPV and radiation response, the American Joint Committee on Cancer (AJCC) created a new staging system in 2016 specifically for patients diagnosed with HPV-positive oropharyngeal cancer to reflect its favorable prognosis compared to those with HPV-negative disease [15]. Notably, many HPV-positive tumors that had been previously categorized as stage IV were significantly “down-staged” to stage II or even stage I cancers. This new staging system has prompted many investigators to question historical treatment paradigms. From a practical standpoint, as long as the cancer is confined to the head and neck, the probability of cure is quite high, eclipsing 90% in many cases [16].

## What are the standard treatment options?

Standard treatment options for localized oropharyngeal cancer, regardless of HPV status, has historically included primary surgery or primary radiation therapy. While some patients with early-stage, low-volume disease can be cured with surgery alone, the vast majority of surgically treated patients will require post-operative radiation (and sometimes even chemotherapy). As a result, primary radiation is increasingly offered as a treatment option. For patients with bulkier disease, concurrent chemotherapy is often added to radiation to increase the potency of treatment. Notably, there are no studies that have shown that either primary surgery or surgery is superior with respect to curing oropharyngeal cancer. Since the prognosis for HPV-positive oropharyngeal cancer is so favorable, attention has focused on optimizing quality of life.

## Is there a role for immunotherapy?

While HPV-positive oropharyngeal cancer is sensitive to most forms of treatment, the role of immunotherapy remains under active investigation. Currently, immunotherapy in the form of checkpoint inhibitors blocking the PD-1/PD-L1 axis is approved for patients with metastatic disease in which the cancer has spread beyond the head and neck region. Another possible indication for immunotherapy is in the recurrent setting when disease returns after initial treatment [17]. However, at present there is no established role for immunotherapy for patients with newly diagnosed HPV-positive oropharyngeal cancer localized to the head and neck. Additionally, vaccine-based therapies are currently under development and being studied in early-stage clinical trials.

## Should a feeding tube be placed prior to treatment?

The role of prophylactic, or preventive, feeding tube placement has significantly dwindled over the years. Given advances in treatment, the ability to preserve swallowing function— with minimally-invasive surgical techniques and with highly conformal methods of radiation delivery— is much more likely than in the past. Published data has shown the routine use of feeding tubes should be avoided because they are associated with long-term atrophy of the swallowing muscles [18]. The exception are patients who present for treatment with significant weight loss (e.g., greater than 10%) even prior to beginning therapy.

## What is de-escalation?

This recognition that HPV-positive oropharyngeal cancer responds favorably to radiation has prompted investigators to suggest that patients with these tumors might be unnecessarily treated with intensive, multi-modality therapy and subjected to the associated toxicity with excessively high radiation doses. As a result, prospective trials have been conducted investigating the role of treatment de-escalation with the goal of reducing side effects, particularly those related to swallowing and salivary function, while maintaining the high rates of cure historically observed [19–22]. The entire premise of de-escalation is based on the idea that by backing off on the intensity of treatment, quality of life can be better optimized without compromising disease control, cure, or survival.

## Who might be eligible for de-escalation?

Patients with newly diagnosed HPV-positive squamous cell carcinoma localized to the head and neck and originating from the oropharynx (i.e., the tonsils, base of tongue, or uvula) are potential candidates for de-escalation. The exact type of de-escalation treatment might vary depending on the extent and location of disease. Notably, published data have also suggested that the favorable impact of HPV on prognosis is particularly strong for those deemed “never smokers” or even those with a minimal smoking history, thereby suggesting that these patients are the most appropriate group for de-escalation [23]. Indeed, numerous studies have shown that tobacco use mitigates the positive prognostic value of HPV and places patients at higher risk for recurrence after treatment [24–26].

## What selection criteria are used for de-escalation?

How to select the most appropriate patients for de-escalation is the active focus of considerable investigation. While prospective studies have demonstrated the feasibility of de-escalation in the setting of HPV-positive oropharyngeal cancer, it is important to recognize the considerable variability in terms of research design, inclusion criteria, and methodology existed across trials [19–22]. Given the lack of standardization, it thus remains exceptionally challenging to categorize how to best select patients for de-escalation. In general, patients with low-volume disease and minimal smoking histories were deemed eligible for de-escalation. It is also notable that some studies have used an adaptive design in which response to induction chemotherapy is used to determine the subsequent radiation dose whereas others opted for proceeding directly to radiation de-escalation with chemotherapy. The major difference in study design, in these situations, makes it difficult to compare strategies. It is likely that integration of additional biological and molecular information both at diagnosis and during treatment will lead to refinements in de-escalation.

## Why is de-escalation being studied?

Historically, the standard non-surgical regimen has consisted of 7 weeks of daily radiation to relatively high doses, often combined with cisplatin chemotherapy. However, this treatment can be difficult to tolerate and also incurs significant post-treatment complications with a significant proportion of patients developing long-term toxicity including swallowing dysfunction, dry mouth, and/or neck stiffness [27]. Unfortunately, these side effects can be severe, life-altering, and permanent. Indeed, the detrimental effect of treatment on quality of life, psychosocial health, and overall functional capacity has been well-established. Since patients with HPV-positive oropharyngeal cancer present at a relatively young age and can potentially survive for decades after treatment, the focus on decreasing long-term complications and optimizing quality of life is particularly relevant. In short, the interest in de-escalation centers on preserving quality of life and function while maintaining the high rates of cure historically observed in these patients.

## What can de-escalation possibly accomplish for patients?

The major side effects of standard treatment with high-dose radiation in both the short- and long-term pertain to difficulty with swallowing—and are primarily related to the incidental exposure of normal tissue to radiation [28]. Indeed, the reported rates of feeding tube dependence, aspiration pneumonia, severe dehydration, and malnutrition are not insignificant among patients completing treatment [29]. The disruption of salivary production, which can often be permanent, also leads to challenges with chewing and speaking [30]. Since studies have shown that the likelihood and severity of side effects increases with radiation dose, it has been hypothesized that by reducing radiation to the normal structures of the head and neck, there will be a consequential decrease in side effects—particularly related to swallowing and salivation—resulting in improved quality of life [31]. Additionally, biological models have shown that reducing radiation should also decrease the incidence of radiation injuries to the bone (osteoradionecrosis), nerves (neuropathy), and soft tissue (fibrosis), among other anatomical structures [32]. Given the contributory role of chemotherapy in potentiating the effects of radiation, it is also hypothesized that reducing the intensity of this modality can decrease these side effects as well. Since chemotherapy has been historically associated with systemic toxicity including bone marrow suppression, ototoxicity, neurotoxicity, and renal dysfunction, the ability to eliminate this modality from treatment paradigms could improve functioning.

## Does this mean that de-escalation can improve quality of life?

Given that the probability of developing most radiation-induced complications can be decreased by reducing the radiation dose exposure to healthy tissue, the potential of de-escalation to improve quality of life for patients undergoing treatment for head and neck cancer is profound. By potentially decreasing toxicity without lowering cure rates, de-escalation for HPV-positive tumors has the potential to improve quality of life for survivors and to allow them to live more functional and productive lives [33]. The potential of de-escalation to reduce psychosocial distress such as symptoms of depression and anxiety is also starting to be realized. In fact, one recent study showed that de-escalation significantly reduced the proportion of survivors dependent on pain medications and opioids after treatment for HPV-positive oropharyngeal cancer [34]. All in all, the alluring potential to improve quality of life is the primary reason that reducing the intensity of treatment through de-escalation is of such strong interest.

## Is it feasible to reduce the radiation dose?

Over the last decade, several prominent prospective trials have been published which have demonstrated promising outcomes with de-escalated radiation regimens using lower than conventionally accepted doses [19–22]. While the design of these trials has varied, these have consistently shown that de-escalated radiation for HPV-positive oropharyngeal carcinoma can maintain the historically high rates of cure while significantly decreasing toxicity and improving quality of life, thus largely validating the premise for which de-escalation was proposed.

One group from the University of California performed a multi-center, phase 2 trial, treating 45 patients with locally advanced HPV-positive oropharyngeal squamous cell carcinoma with 2 cycles of induction chemotherapy given 21 days apart, followed by de-escalated radiation [19]. At 2-years, the reported rates of disease control and overall survival were 92% and 98%, respectively, which compared favorably to historical controls treated without de-escalation. As importantly, the incidence of gastrostomy-tube dependence rate at 6-months post-radiation and severe swallowing dysfunction was zero. A prospective analysis of endpoints related to quality of life and pre- and post-therapy swallow studies showed that de-escalation dramatically improved function with respect to every variable analyzed including weight loss, depression, and opioid usage compared to contemporary control subjects who opted not to be treated with de-escalation [34]. A survey of perspectives and attitudes of subjects treated on the University of California de-escalation trial showed that nearly all patients were satisfied with their decision and any regret was nearly non-existent [35].

While other groups have helped validate the paradigm of induction chemotherapy prior to de-escalated radiation for HPV-positive oropharyngeal cancer, others have investigated different approaches. For instance, the use of concurrent chemoradiation using de-escalated radiation with weekly cisplatin has also been shown to lead to excellent outcomes [36]. As a result, when de-escalated radiation is considered in the setting of chemoradiation, two different strategies have been proposed, one utilizing concurrent chemotherapy and other with induction chemotherapy.

## Is it possible to modify the chemotherapy or eliminate it altogether?

The purpose of chemotherapy, when it is given together with radiation, is to generally make radiation more effective. In the setting of head and neck cancer, it is considered exclusively a “radiosensitizer.” Studies have demonstrated a lack of systemic benefit associated with chemotherapy in controlling potential deposits of occult, micrometastatic disease has been shown specifically for patients with HPV-positive squamous cell carcinoma [37]. Given the exquisitely sensitivity of HPV-positive oropharyngeal cancer to radiation, a logical question is whether chemotherapy is needed. Since biological models have suggested that the addition of chemotherapy is equivalent to about 3–5 extra radiation sessions, a strategy of intensifying treatments seems to be paradoxical to the premise of de-escalation [38]. One way to approach this dilemma is to change the way in which chemotherapy is delivered. For instance, studies have now shown that weekly delivery of smaller amounts of cisplatin might be just as effective and better tolerated as administering the chemotherapy every 3 weeks using larger doses as traditionally done [39]. While attempts have been made to replace cisplatin with the targeted systemic agent, cetuximab, the results of prospective trials have suggested that this may lead to inferior outcomes [40–42]. These results raise caution at how efforts to explore de-escalation should be conducted in the future, as they clearly suggest that cisplatin still has an important role in the treatment of HPV-positive oropharyngeal cancer. How to reconcile these findings with ongoing attempts at de-escalation requires thoughtful deliberation. The logical question revolves around which patients actually benefit from the addition of cisplatin to radiation; and conversely, which patients would it be acceptable to avoid cisplatin? Given that prospective studies have varied widely in terms of selection with respect to such factors as tumor volume, disease bulk, and smoking history, among others, there is currently no consensus that exists with respect to these questions. However, it is clear that efforts to better understand selection criteria using both traditional factors such as AJCC stage and evolving molecular information are imperative so that de-escalation can be refined. Studies analyzing whether immunotherapy can be utilized as an alternative are also ongoing [43].

The evidence in favor of radiation alone for appropriately selected patients with HPV-positive oropharyngeal carcinoma is provocative. Based on historic data from the University of California, Davis and the Princess Margaret Hospital showing that radiation alone can be curative for many patients with HPV-positive oropharyngeal carcinoma, investigators from Japan recently published a phase 2 trial showing positive outcomes and a survival of 100% [44–46]. In a phase II study, investigators from the University of North Carolina reported on a small subset of patients with lower tumor volumes who were treated with de-escalated radiation alone with excellent outcomes [47]. While NRG HN02 showed that the addition of concurrent cisplatin to de-escalated radiation reduced the 2-year local failure rate from 9 to 3% it was unclear which patients benefited the most [48]. When the 2-year disease control and overall survival rates were analyzed, no differences were observed between patients treated by de-escalated radiation with or without chemotherapy. These studies suggest that some patients with HPV-positive oropharyngeal squamous cell carcinoma can be treated with de-escalation using radiation alone and achieve excellent outcomes. However, further work to better identify selection criteria to assist in the stratification of treatment is needed.

## Is transoral robotic surgery (TORS) considered de-escalation?

Minimally invasive operative techniques using TORS has also been proposed as a means of de-escalating treatment for HPV-positive oropharyngeal cancer. While the treatment by itself is not considered de-escalation since it is a type of surgery, it is increasingly clear that TORS can serve as the first step in facilitating de-escalation in some cases. In select patients with low volume disease, TORS, combined with a surgical neck dissection may actually serve as a replacement for radiation, eliminating the need for further treatment altogether [49]. While TORS has been shown to be a reasonable initial option for some patients with HPV-positive oropharyngeal cancer, it must be recognized that a substantial proportion of patients will require radiation post-operatively anyhow [50]. However, studies have now been published showing that lower than possible radiation doses or smaller target volumes can be delivered after TORS—thereby leading to improvements in quality of life for patients with HPV-positive oropharyngeal cancer [51–53]. For instance, the Eastern Cooperative Oncology Group conducted a phase II study randomizing patients to 50–60 Gy after TORS and reported 2-year progression-free survival rates of 95% and 96%, respectively [52]. Similarly, results from a phase II data from Mayo Clinic reporting on the use of 30–36 Gy in 20 fractions using twice-daily treatments in combination with chemotherapy showed 2-year progression-free survival and overall survival of 91% and 99%, respectively. As impressively, the rates of grade 3 or worse toxicity at 1- and 2- years post-RT were 0%, and 0%, respectively [53]. While these approaches have largely corroborated the utility of TORS in allowing for de-escalation, it is still unclear which selection criteria should be used to optimize outcomes, especially when deciding between surgical and non-surgical treatment upfront. Similarly, strategies for adjuvant therapy for patients treated by TORS continue to evolve, and traditional indications for the use of post-operative radiation or chemoradiation have been questioned in the setting of HPV-positive oropharyngeal cancer [54]. Enthusiasm for the use of TORS also may have been dampened by the results of the ORATOR trial which randomized patients with newly diagnosed HPV-positive oropharyngeal cancer to either initial TORS or to primary radiation [55]. While survival and cure rates were the same between the 2 arms, patients randomized to TORS had significantly decreased swallowing function at 1-year.

## Why is de-escalation so popular?

De-escalation has ushered in an exciting new era in the management of head and neck cancer. Indeed, for HPV-positive oropharyngeal cancer, de-escalation is considered to be a form of precision medicine—using the biological characteristics of the tumor to drive treatment decision-making. By moving away from a “one size fits all” approach to disease management, de-escalation has the potential to customize treatment to the fullest for individuals. The popularity of this approach has been driven by the increasing recognition that HPV-related oropharyngeal cancer is exquisitely sensitive to radiation, as well as the increased desire of patients to avoid side effects.

It is likely that advances in de-escalation will use biological information from the tumor itself to guide treatment. As an example, investigators from Memorial Sloan Kettering have utilized hypoxia monitoring using novel radiotracers to select patients for radical de-escalation of radiation dose. Utilizing fluoromisonidazole-positron emission tomography (F-MISO-PET) to image hypoxia during radiation, they showed that radiation dose reduction to 30 Gy for tumors with no pretreatment hypoxia or in whom hypoxia had resolved within the first 2 weeks of initiating radiation was feasible [56]. In their phase II study, 158 patients first underwent surgical removal of disease at their primary site, but not of gross disease in the neck [57]. A baseline ^18^F-fluoromisonidazole positron emission tomography scan was used to measure tumor hypoxia and was repeated 1–2 weeks mid-treatment. Patients with nonhypoxic tumors received 30 Gy (3 weeks) with chemotherapy, whereas those with hypoxic tumors received standard chemoradiotherapy to 70 Gy (7 weeks). With a median follow-up time of 38 months, the 2-year PFS was 94% and overall survival was 100%, respectively for the 30 Gy cohort suggesting that dramatic reductions in dose can be feasibly used with the incorporation of hypoxia-based risk-adaption.

## Is de-escalation ready to become standard treatment?

Given the preponderance of evidence attesting to the sensitivity of HPV-positive oropharyngeal cancer to radiation, a tremendous amount of attention has focused on investigating whether patients with locally advanced HPV-positive oropharyngeal cancer should be treated differently than those with HPV-negative tumors. The concept of de-escalation encompasses a variety of different strategies intended to make treatment gentler through a reduction in radiation, alteration in chemotherapy regimens, and/or elimination of either modality altogether. However, how to best offer this approach to patients is uncertain, as various methods have been described; and the question of whether de-escalation is even ready for use outside of a clinical trial is hotly debated.

While the overriding goal of de-escalation is to maintain the high survival rates associated with traditional approaches while reducing the incidence of both short- and long-term toxicity by lessening the intensity of treatment, it is still not considered standard of care. This is because the published data is still relatively preliminary and complicated by such factors as the variability in treatment, inclusion criteria, and follow-up. However, the reality of clinical decision-making for HPV-positive oropharyngeal cancer has evolved to the point where patients are now routinely demanding de-escalated radiation. Although this strategy is seemingly well-supported by the depth and breadth of data that has been published reporting on outcomes of de-escalation, attempts to replicate these outside of a clinical trial should be avoided. Nonetheless, findings from a recent patterns of care analysis demonstrated that de-escalated radiation has become increasingly offered to patients with HPV-positive oropharyngeal cancer as standard treatment—an undeniable fact attesting to the popularity of this concept [58].

## What happens after treatment?

Adjusting to a “new normal” and learning to cope with an umbrella of physical and emotional changes after cancer treatment is essentially what defines survivorship. This is particularly important in the setting of HPV-positive oropharyngeal cancer due to the spectrum of physical and psychosocial changes that frequently accompany the diagnosis and treatment of these malignancies. Since patients were treated with a combination of surgery, chemotherapy, and/or radiation therapy, the functional and quality of life implications associated with these modalities must be considered. In addition to regular follow-up visits with providers, patients are encouraged to maintain contact with their dentist, swallow therapist, and nutritionist, as the need arises. For patients with lymphedema of the face and/or neck, referral to a physical therapist may be beneficial.

## What follow-up tests are recommended?

The exact recommendations for follow-up are typically made on a case-by-case basis, depending on such factors as one’s specific treatment and functional well-being. In general, new post-treatment baseline imaging is obtained at 1 and 3 months, respectively, for patients treated by primary surgery and radiation therapy, respectively. For those with no evidence of clinical disease thereafter, the role of further surveillance imaging is controversial and not should be avoided in the absence of any new symptoms [59, 60]. However, regular endoscopic examination and palpation of the neck should be performed at least every 3 months for the first 2 to 3 years after treatment. Given that many patients have lost weight during treatment, regular blood tests should also be performed to optimize function. Since hypothyroidism occurs in a third to nearly half of patients who receive radiation to their neck, often as a late sequela, regular evaluation for thyroid dysfunction by measuring thyrotropin and thyroxine is recommended. Lastly, the need for patients to be self-vigilant cannot be overstated. Early detection of new symptoms including sore throat, ear pain, neck mass, or other concerning findings should prompt early return to the oncology team for further evaluation.

## What follow-up tests are recommended?

Continued efforts to better refine selection criteria as well as to dynamically monitor treatment response will define the evolution of treatment for HPV-positive oropharyngeal cancer, particularly with respect to de-escalation. At present, the only factor (other than AJCC cancer stage) that is used for risk stratification is smoking history. Future advances in de-escalation will need to incorporate a combination of clinical, radiological, and biological data—helping to apply principles of precision medicine to this approach. For instance, considerable interest has arisen in using HPV DNA levels from the blood, obtained before, during, and after treatment, to monitor de-escalation [61]. Another approach involves using special imaging techniques, often combined with machine learning and/or artificial intelligence algorithms, to predict response to de-escalation [62]. While these approaches hold promise for the future, exactly how to utilize such strategies remain uncertain and the subject of ongoing research.

## Conclusion

The recognition that HPV-positive oropharyngeal cancer represents a uniquely different disease has ushered in a new era of treatment focused on individualization of care. While both surgery and radiation have historically been used as curative modalities in this setting, de-escalated treatment has now been established as an attractive option in the management of HPV-positive oropharyngeal cancer due to its demonstrated ability to preserve quality of life and functioning while maintaining high rates of cure. Yet questions continue to persist regarding how to best achieve de-escalation. While some patients can likely be effectively treated with de-escalated radiation alone, it is possible that others with higher-risk disease might benefit from the addition of chemotherapy to de-escalated radiation. However, these paradigms continue to evolve as studies contribute to an improved understanding of HPV-related oropharyngeal cancer. While enthusiasts argue that the data robustly supports the integration of de-escalation into contemporary practice; skeptics point out that the published data is still relatively preliminary and makes it difficult to assert definitive recommendations. Based on the emerging evidence, as well as on the explosion in interest from patients and physicians alike, well-designed clinical trials with associated translational studies are urgently needed to better refine selection criteria for de-escalation and to stratify patients with newly diagnosed oropharyngeal cancer into the appropriate means of treatment. Given the increasing incidence of this disease, the importance of this research cannot be overstated.

## Data Availability

No datasets were generated or analysed during the current study.
